# Houselessness and syringe service program utilization among people who inject drugs in eight rural areas across the USA: a cross-sectional analysis

**DOI:** 10.1186/s12954-023-00892-w

**Published:** 2023-10-26

**Authors:** April M. Ballard, Dylan Falk, Harris Greenwood, Paige Gugerty, Judith Feinberg, Peter D. Friedmann, Vivian F. Go, Wiley D. Jenkins, P. Todd Korthuis, William C. Miller, Mai T. Pho, David W. Seal, Gordon S. Smith, Thomas J. Stopka, Ryan P. Westergaard, William A. Zule, April M. Young, Hannah L. F. Cooper

**Affiliations:** 1 Rollins School of Public Health, Emory University, 1518 Clifton Road, Atlanta, GA 30322 USA; 2 School of Public Health, Georgia State University, 140 Decatur Street SE, Atlanta, GA 30303 USA; 3 School of Medicine, West Virginia University, Morgantown, WV USA; 4 Office of Research, University of Massachusetts Chan Medical School-Baystate, Springfield, MA USA; 5 Gillings School of Global Public Health, University of North Carolina at Chapel Hill, Chapel Hill, NC USA; 6 School of Medicine, Southern Illinois University, Carbondale, IL USA; 7 Department of Medicine, Oregon Health & Science University, Portland, OR USA; 8 College of Public Health, The Ohio State University, Columbus, OH USA; 9 Department of Medicine, University of Chicago, Chicago, IL USA; 10 School of Public Health and Tropical Medicine, Tulane University, New Orleans, LA USA; 11 School of Medicine, Tufts University, Boston, MA USA; 12 School of Medicine and Public Health, University of Wisconsin-Madison, Madison, WI USA; 13 RTI International, Research Triangle Park, NC USA; 14 College of Public Health, University of Kentucky, 111 Washington Avenue, Lexington, KY 40536 USA

**Keywords:** Houselessness, Syringe service programs, Harm reduction, Rural areas, Healthcare access

## Abstract

**Background:**

Research conducted in urban areas has highlighted the impact of housing instability on people who inject drugs (PWID), revealing that it exacerbates vulnerability to drug-related harms and impedes syringe service program (SSP) use. However, few studies have explored the effects of houselessness on SSP use among rural PWID. This study examines the relationship between houselessness and SSP utilization among PWID in eight rural areas across 10 states.

**Methods:**

PWID were recruited using respondent-driven sampling for a cross-sectional survey that queried self-reported drug use and SSP utilization in the prior 30 days, houselessness in the prior 6 months and sociodemographic characteristics. Using binomial logistic regression, we examined the relationship between experiencing houselessness and any SSP use. To assess the relationship between houselessness and the frequency of SSP use, we conducted multinomial logistic regression analyses among participants reporting any past 30-day SSP use.

**Results:**

Among 2394 rural PWID, 56.5% had experienced houselessness in the prior 6 months, and 43.5% reported past 30-day SSP use. PWID who had experienced houselessness were more likely to report using an SSP compared to their housed counterparts (adjusted odds ratio [aOR] = 1.24 [95% confidence intervals [CI] 1.01, 1.52]). Among those who had used an SSP at least once (*n* = 972), those who experienced houselessness were just as likely to report SSP use two (aOR = 0.90 [95% CI 0.60, 1.36]) and three times (aOR = 1.18 [95% CI 0.77, 1.98]) compared to once. However, they were less likely to visit an SSP four or more times compared to once in the prior 30 days (aOR = 0.59 [95% CI 0.40, 0.85]).

**Conclusion:**

This study provides evidence that rural PWID who experience houselessness utilize SSPs at similar or higher rates as their housed counterparts. However, housing instability may pose barriers to more frequent SSP use. These findings are significant as people who experience houselessness are at increased risk for drug-related harms and encounter additional challenges when attempting to access SSPs.

**Supplementary Information:**

The online version contains supplementary material available at 10.1186/s12954-023-00892-w.

## Background

Injection drug use continues to be a significant public health concern, with recent global estimates indicating that 23% and 10% of new hepatitis C virus (HCV) and human immunodeficiency virus (HIV) infections are attributable to injection drug use, respectively [1, 2]. Rural areas are disproportionately impacted by HCV and HIV infections in many countries due, in part, to a higher prevalence of injection drug use and limited access to harm reduction services in these regions [3–11]. In response to these disparities, syringe services programs (SSPs) are expanding to rural areas [5, 7, 8, 10, 12]. However, coverage remains varied and inequities in access among people who inject drugs (PWID) threaten SSPs’ ability to curb drug-related epidemics [13–16].

Drug-related epidemics have been expanding to rural areas across the globe for several decades [5], including in the USA [3], Canada [7] and Australia [17]. The rise of prescription opioids and increasing availability of heroin and methamphetamine have contributed to the spread of these epidemics, which have historically been associated with urban areas, to more rural regions over time [3, 11, 18–23]. Some countries have begun successfully operating SSPs in rural areas to reduce the spread of bloodborne infections [7, 15, 24, 25], though there are many barriers (e.g., funding, criminalization of substance use, stigma, local policy) to their widespread implementation and use [26–28]. The expansion of rural SSPs in the USA has been particularly significant [13, 15, 24, 29, 30]. In 2022, there were more than 100 rural SSPs in operation [29, 31] compared to only 30 in 2013 [29]. However, amidst this rapid expansion of SSPs to rural areas, research to assess utilization and barriers to access among rural PWID has been limited [13, 14, 32, 33].

Research conducted in cities has identified housing instability among PWID as a factor both that exacerbates vulnerability to drug-related harms and also that can significantly impede SSP utilization [34–39]. In the USA, urban PWID experience high rates of houselessness [39–42]. A study conducted in 23 cities found that 68% of PWID had experienced houselessness in the last year [40]. Urban PWID who experience houselessness have been shown to have perceived and measured reductions in healthcare access [43–46]. Houselessness has also been identified as an important risk factor for acquisition of bloodborne infections [2, 38, 39, 47], and many HIV outbreaks have occurred across multiple countries over the last decade among PWID who were experiencing homelessness [48–53]. Notably, some studies have found that PWID who experience houselessness in the urban USA access harm reduction services more than their housed counterparts [35, 54], yet sharing injection equipment and non-fatal overdoses are more likely among this population [35, 38, 55–57]. Understanding and addressing inequities in SSP access and utilization for PWID who are unstably housed therefore must be a critical component of efforts to mitigate drug-related epidemics.

Though houselessness is expanding to the rural USA in parallel with, yet distinct from, the opioid epidemic, few studies have explored the relationship between houselessness and SSP use among rural PWID [14, 32, 33]. Rural houselessness counts are likely underestimated due to underreporting and gaps in data. Still, the 2022 Point-In-Time Count estimates that approximately 18% of all people experiencing houselessness in the USA were located in rural areas. Rural areas also experienced the largest overall percentage increase in houselessness between 2020 and 2022 compared to urban and suburban areas [58]. Structural inequities—including economic disparities, lack of employment opportunities and inadequate infrastructure for public housing and supportive services—are the main drivers of growing houselessness in rural areas [59, 60]. At the individual level, houselessness is likely exacerbated by drug use in rural communities that have been disproportionately impacted by the opioid crisis [61–64].

This study expands upon USA-based research on houselessness and SSPs by examining this relationship among PWID from eight rural areas across 10 states. Specifically, we examine the relationship of houselessness to any recent SSP use and also to the frequency of SSP use among those who had utilized an SSP to get syringes or needles at least once in the prior 30 days. Findings from this study can provide insights into SSP utilization in rural areas and be used to inform the development of targeted strategies to address inequities in access among PWID.

## Methods

### Study design, sample and data collection

This study analyzed data generated by the Rural Opioid Initiative (ROI), a multistate study that collected data on demographics, drug use, drug-related harms (e.g., HCV and HIV infections, non-fatal overdose) and healthcare use among rural people who use drugs, regardless of whether they inject [3]. The ROI enrolled participants for a cross-sectional survey from eight rural sites spanning 10 states, including Kentucky, Wisconsin, New England (i.e., Massachusetts, Vermont and New Hampshire), Illinois, West Virginia, Oregon, Ohio and North Carolina. Participants across all study studies were recruited from January 2018 to March 2020 using modified chain-referral sampling based on respondent-driven sampling (RDS) methods [3, 65, 66]. This approach relied on waves of peer-to-peer recruitment where referral chains were tracked and chain structure is used in analyses [66].

Eligibility criteria were standardized across research projects with two exceptions. At six of the eight sites, participants had to: (1) be at least 18 years old; (2) self-report any injection drug use or non-injection opioid use in the prior 30 days ‘to get high’; and (3) live in the site’s catchment area. Variations to these criteria were used in Illinois and Wisconsin, where individuals aged 15–17 were eligible because the projects were embedded in organizations that provide services to adolescents, and in Wisconsin, where only clients with a history of injection drug use were included. Surveys were conducted in a private space using multiple methods across study sites: Five of the sites used audio computer-assisted self-interviews, two used computer-assisted self-interviews, and one site used computer-assisted personal interviews. Participants received $40–60 for their participation, depending on the site. Additional data collection and management details for the ROI are published elsewhere [3].

We conducted two analyses. First, we examined the relationship between houselessness and any SSP use. among people who had recently injected drugs. The ROI survey asked all participants, ‘Have you ever injected drugs to get high?’ Participants who reported injection drug use in their lifetime were then asked, ‘When did you last inject drugs to get high?’ Those who reported a date within the past 30 days were included in our analytic sample.

Second, we evaluated the association between houselessness and frequency of SSP use. Participants who reported injecting drugs in the prior 30 days were also asked, ‘During the last 30 days, where have you gotten syringes or needles?’ Multiple answers were provided (e.g., a syringe or needle exchange program in person, from someone else who got them from a syringe or needle exchange program, farm supply store, pharmacy) and participants were able to select all that applied. Those who reported getting syringes or needles from an SSP were included in our analytic sample.

### Measures

The primary independent variable of interest in this study was experiencing houselessness in the prior 6 months. All ROI participants were asked, ‘Have you been homeless in the past 6 months? “Homeless” means you were living from place to place, “couch-surfing,” on the street, in a car, park, abandoned building, squat or shelter.’ Participants could respond ‘yes,’ ‘no,’ and ‘don’t know.’ We use the term ‘houseless’—as opposed to the government standard term ‘homeless’—throughout to emphasize that individuals lack a permanent physical structure to live in, but do not lack personal community.

There were two dependent variables of interest related to SSP use: (1) any SSP use in the prior 30 days and (2) the frequency of SSP use in the prior 30 days. Any use was derived from the select all survey question described above, ‘During the last 30 days, where have you gotten syringes or needles?’ The frequency of SSP use was derived from responses to a survey question that asked participants who reported any SSP use in the prior 30 days, ‘How many times in the past 30 days did you get new syringes or needles, cottons or cookers from a syringe or needle exchange program?’ We created a categorical variable by discretizing the original numeric responses into four categories (i.e., once, twice, three times, or four times or more in the prior 30 days) because the distribution was skewed and some SSPs in study areas were only open once a week (i.e., approximately four times per month).

Notably, the recall periods for houselessness and SSP use differed in this study. This is a limitation since we do not know the exact time within the 6-month period when houselessness occurred, nor do we know whether houselessness was persistent throughout the duration. This is acknowledged and incorporated into our study’s scope. Specifically, all interpretations are grounded in the assumption that experiencing houselessness in the prior 6 months either preceded or coincided with the prior 30 days (i.e., the SSP use recall period). Furthermore, while participants may not have been experiencing houselessness during the 30-day time frame, experiencing houselessness at any time is likely indicative of housing instability, which has been shown to be associated with drug-related harms [34, 67–69] and access to health services [70, 71].

### Analyses

We used descriptive statistics to summarize houselessness and participant characteristics for the entire sample, and by any SSP use and frequency of use in the prior 30 days. We assessed associations between any SSP use and participant characteristics using bivariate logistic regressions, including random effects to account for clustering due to the ROI RDS approach and site of enrollment. Then, we used multivariable binomial and multinomial logistic regression to estimate the association between houselessness and any SSP use and the frequency of SSP use, separately. Specifically, we used the *lme4* package [72] in R Studio v4.0.5 [73] to conduct multivariable binomial logistic regression analyses to assess the relationship between houselessness and any SSP use, and the *mclogit* package [74] to conduct multivariable multinomial logistic regression to assess the relationship between houselessness and the frequency of SSP use. We used a multinomial model because the effect of houselessness on frequency of SSP use was not constant, violating the proportional odds assumption for ordinal regression models. All models included random effects for RDS chains and study site of enrollment. For each set of analyses, we considered covariates for inclusion based on previous literature and a priori hypotheses [32, 34, 36, 56, 64, 75], which included demographic characteristics (e.g., gender, age, race, educational attainment), entitlements (e.g., food pantry use) and type of drugs used to get high in the last 30 days (e.g., heroin, methamphetamine). Potential confounders with *p* values ≤ 0.10 in bivariate analyses were included in models.

## Results

**Fig. 1:**
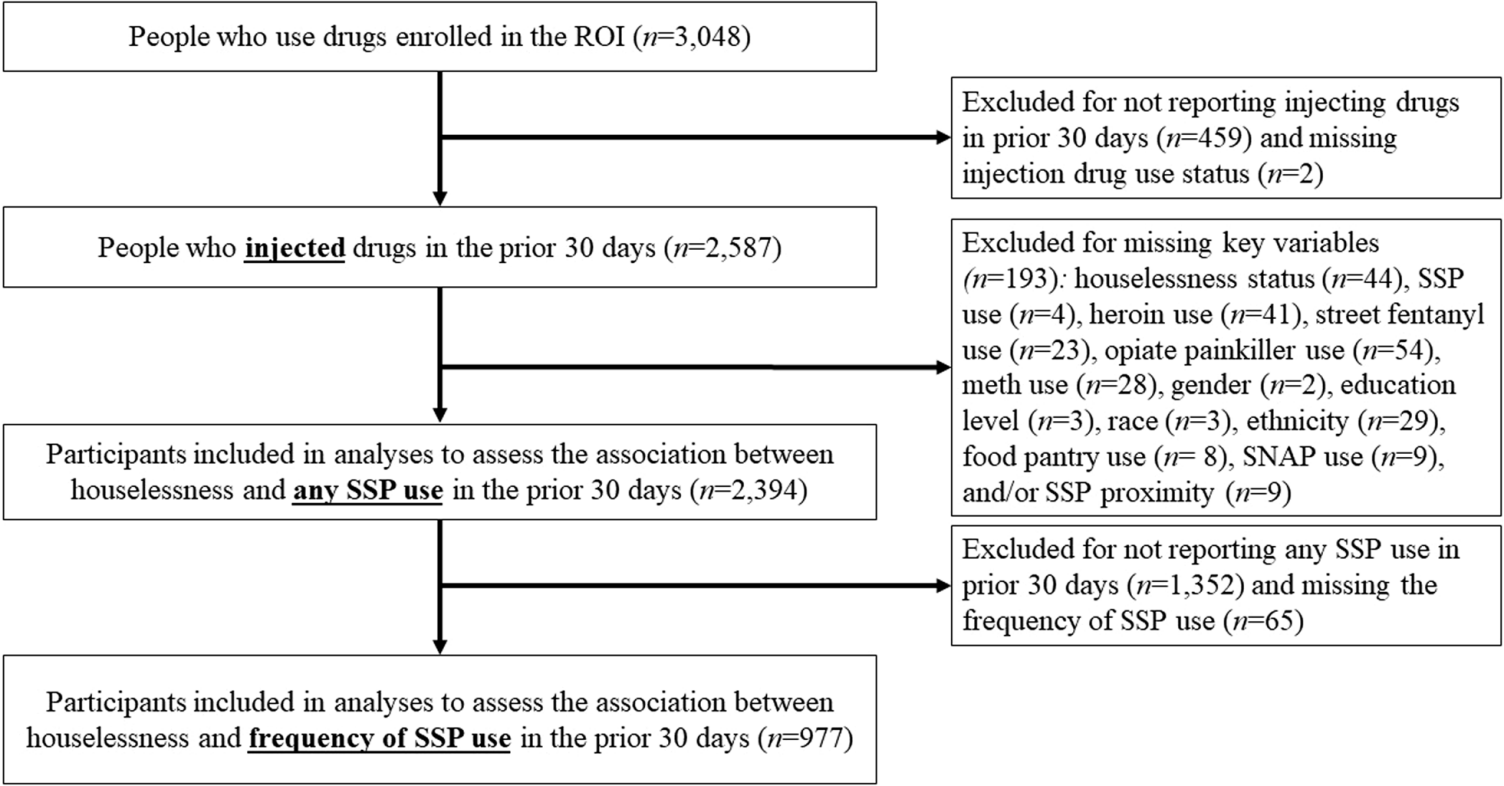
Analytic sample flow diagram

The ROI enrolled 3048 participants, 84.9% (*n* = 2587) of whom reported injecting drugs in the prior 30 days. One hundred ninety-three other participants who lacked data on key variables were excluded from the analytic sample to assess the association between houselessness and any SSP use, and 65 were excluded from the sample to assess the relationship between houselessness and frequency of SSP use. Figure 1 provides a flow diagram of the analytic sample.

### Any SSP use

Our final sample of PWID included 2394 participants, who were predominantly white (85.6%), men (57.3%) and high school graduates (78.6%) (Table 1). Participants were 36 years old on average (standard deviation [SD] = 10). The most prevalent drugs used were methamphetamine (80.5%) and heroin (72.7%). Participants reported varying proximity to SSPs: 40% were within walking distance, 34.7% were less than a 30-min drive, and 11.6% were more than a 30-min drive. Many (10.6%) did not know where the closest SSP was located. Most participants (56.5%) had experienced houselessness in the prior 6 months and less than half (43.5%) reported getting syringes or needles from an SSP in the prior 30 days, though this varied across study sites (Additional file 1: Table S1). The rate of houselessness was the highest among PWID in Oregon (68.2%) and the lowest among those in Kentucky (38.1%). SSP use was the greatest among Wisconsin-based PWID (63.2%) and the smallest among West Virginia-based PWID (10.7%). Unadjusted associations between houselessness, covariates and SSP use are presented in Table 1. Results of adjusted analyses are given in Table 2 and are expanded upon below.

**Table 1:** Characteristics of people who injected drugs enrolled in the Rural Opioid Initiative by self-report SSP use in the prior 30 days (*n* = 2394)

Characteristic	Total	Used an SSP^b^	Did not use an SSP^b^	Unadjusted^c^	
	*n* (%)	*n* (%)	*n* (%)	OR	(95% CI)
	2394 (100.0)	1042 (43.5)	1352 (56.5)		
Experienced houselessness^a^	1342 (56.1)	620 (59.5)	723 (53.5)	**1.26**	**(1.04, 1.54)**
*Gender*					
Man	1371 (57.3)	598 (57.4)	773 (57.2)	Ref.	–
Woman	1010 (42.2)	438 (42.0)	572 (42.3)	0.98	(0.81, 1.19)
Other	13 (0.5)	6 (0.6)	7 (0.5)	0.64	(0.18, 2.22)
Age (years; mean [sd])	36 (10)	35 (9)	36 (10)	**0.98**	**(0.97, 0.99)**
*Race*					
White	2050 (85.6)	862 (82.7)	1188 (87.9)	Ref.	–
Black	63 (2.6)	19 (1.8)	44 (3.3)	**0.49**	**(0.26, 0.92)**
Native American	180 (7.5)	116 (11.1)	64 (4.7)	**2.05**	**(1.38, 3.04)**
Other	101 (4.2)	45 (4.3)	56 (4.1)	0.98	(0.61, 1.56)
*Ethnicity*					
Hispanic	80 (4.2)	33 (3.2)	47 (3.5)	0.71	(0.42, 1.20)
Graduated high school	1882 (78.6)	839 (80.5)	1043 (77.1)	1.16	(0.92, 1.46)
Received SNAP^a^	1336 (55.8)	554 (53.2)	812 (60.1)	**0.81**	**(0.66, 0.99)**
Received food from food pantry^a^	1262 (52.7)	514 (49.3)	748 (55.3)	0.93	(0.76, 1.13)
*Drug use*^b^					
Methamphetamine/crystal	1926 (80.5)	889 (85.3)	1037 (76.7)	1.22	(0.91, 1.63)
Heroin	1740 (72.7)	780 (74.9)	960 (71.0)	**1.66**	**(1.31, 2.09)**
Street fentanyl	940 (39.3)	400 (38.4)	540 (39.9)	**1.68**	**(1.34, 2.10)**
Opiate painkillers	1340 (56.0)	553 (53.1)	787 (58.2)	**0.80**	**(0.66, 0.98)**
*Proximity to SSP*					
Walking distance^d^	958 (40.0)	507 (48.7)	451 (33.4)	Ref.	–
< 30-min drive	830 (34.7)	436 (41.8)	394 (29.1)	1.01	(0.80, 1.26)
> 30-min drive	277 (11.6)	82 (7.9)	195 (14.4)	**0.42**	**(0.30, 0.59)**
No program reasonably close	76 (3.2)	12 (1.2)	64 (4.7)	**0.25**	**(0.13, 0.51)**
Don’t know how close an SP is	253 (10.6)	5 (0.5)	248 (18.3)	**0.03**	**(0.01, 0.08)**

Bold text indicates statistically significant at *p *< 0.05

*SSP* Syringe service program, *OR* odds ratio, *CI* confidence interval, *SD* standard deviation, *SNAP* supplemental nutrition assistance program

^a^Reference period: prior 6 months

^b^Reference period: prior 30 days

^c^Bivariate binomial logistic regression results, controlling for RDS and study site clustering

^d^Includes brick and mortar exchanges and mobile exchanges that come near where the participant lives

**Table 2:** Multivariable binomial logistic regression associations between houselessness in the prior 6 months and using an SSP in the prior 30 days among Rural Opioid Initiative PWID (*n* = 2394)

Characteristic	aOR	(95% CI)
Experienced houselessness^a^	**1.24**	**(1.01, 1.52)**
Age (years)	**0.99**	**(0.98, 1.00)**
*Race*		
White	Ref.	–
Black	0.53	(0.27, 1.02)
Native American	**1.74**	**(1.16, 2.62)**
Other	1.02	(0.63, 1.66)
Received SNAP^a^	0.85	(0.69, 1.04)
*Drug Use*^*b*^		
Heroin	**1.51**	**(1.16, 1.96)**
Street fentanyl	**1.36**	**(1.06, 1.74)**
Opiate painkillers	**0.68**	**(0.55, 0.85)**
*Proximity to SSP*		
Walking distance	Ref.	–
< 30-min drive	0.98	(0.78, 1.23)
> 30-min drive	**0.45**	**(0.31, 0.63)**
No program reasonably close	**0.25**	**(0.12, 0.52)**
Don’t know how close an SSP is	**0.03**	**(0.01, 0.09)**

Bold indicates statistically significant at *p* < 0.05

*PWID* People who inject drugs, *aOR* adjusted odds ratios, *CI* confidence interval, *SNAP* supplemental nutrition assistance program, *SSP* syringe service program

^a^Reference period: prior 6 months

^b^Reference period: prior 30 days

In adjusted analyses, PWID who had experienced houselessness in the prior 6 months were 24% more likely to report getting syringes or needles from an SSP in the prior 30 days compared to those who had not experienced houselessness (95% confidence interval [CI] 1.01, 1.52—Table 2). Self-reported proximity to an SSP was also associated with SSP use in the prior 30 days. Specifically, those who lived more than 30 min by car from an SSP were less likely to report using one in the prior 30 days compared to those who lived within walking distance (adjusted odds ratio [aOR] = 0.45 [95% CI 0.31, 0.63]). Participants who did not live reasonably close to an SSP or did not know where the nearest SSP was located were also less likely to use an SSP in the prior 30 days compared to those who lived within walking distance (aOR = 0.25 [95% CI 0.12, 0.52] and aOR = 0.03 [95% CI 0.01, 0.09], respectively). Conversely, PWID who lived less than 30 min by car from an SSP were just as likely to use an SSP in the prior 30 days compared to those who lived in walking distance (aOR = 0.98 [95% CI 0.78, 1.23]).

### Frequency of SSP use

The final analytic sample to assess the association between experiencing houselessness and the frequency of SSP use included the 977 participants who reported using an SSP at least once in the prior 30 days. Participant demographics were comparable to the full sample of PWID: predominantly white (83.2%), men (56.5%), high school graduates (81.2%) and 35 years old, on average (SD = 9). Methamphetamine (84.9%) and heroin (75.9%) were the most prevalent drugs (Table 3). Those who used an SSP at least once in the prior 30 days were largely proximal to an SSP: 48.1% of PWID were within walking distance and 42.2% were within 30 min by car. Most (59.6%) had experience houselessness in the prior 6 months. The frequency of SSP use in the prior 30 days varied: 23.8% had gotten new injection equipment from an SSP once, 22.0% had twice, 14.3% had three times, and 39.8% had four or more times. The frequency of SSP use by ROI study site is presented in Additional file 1: Table S2. Unadjusted associations between houselessness, covariates and the frequency of SSP use can be found in Table 4. Results from adjusted analyses are presented in Table 5 and are described below.

**Table 3:** Characteristics of people who injected drugs *and* used an SSP at least once in the prior 30 days enrolled in the Rural Opioid Initiative (*n* = 977)

Characteristics	Frequency of SSP use in prior 30 days				
	Total	Once	Twice	Three times	Four or more times
	*n* (%)	*n* (%)	*n* (%)	*n* (%)	*n* (%)
	977 (100.0)	233 (23.8)	215 (22.0)	140 (14.3)	389 (39.8)
Experienced houselesssness^a^	582 (59.6)	146 (62.7)	133 (61.9)	97 (69.3)	206 (53.0)
*Gender*					
Man	552 (56.5)	133 (57.1)	122 (56.7)	73 (52.1)	224 (57.6)
Woman	420 (43.0)	99 (42.5)	92 (42.8)	64 (45.7)	165 (42.4)
Other	5 (0.5)	1 (0.4)	1 (0.5)	3 (2.1)	0 (0.0)
Age (years; mean [sd])	35 (9)	36 (9)	35 (9)	33 (9)	35 (9)
*Race*					
White	813 (83.2)	198 (85.0)	188 (87.4)	116 (82.9)	322 (79.9)
Black	17 (1.7)	2 (0.9)	4 (1.9)	0 (0.0)	12 (3.0)
Native American	105 (10.7)	19 (8.2)	15 (7.0)	16 (11.4)	56 (13.9)
Other	42 (4.3)	14 (6.0)	8 (3.7)	8 (5.7)	13 (3.2)
*Ethnicity*					
Hispanic	31 (3.2)	8 (3.4)	4 (1.9)	3 (2.1)	16 (4.1)
Graduated high school	793 (81.2)	194 (83.3)	187 (87.0)	116 (82.9)	296 (76.1)
Received SNAP^b,^	524 (53.6)	127 (54.5)	112 (54.4)	71 (50.7)	209 (53.7)
Received food from food pantry^b^	492 (50.4)	114 (48.9)	112 (52.1)	72 (51.4)	194 (49.9)
*Drug use*^*b*^					
Methamphetamine/crystal	829 (84.9)	205 (88.0)	179 (83.3)	122 (87.1)	333 (83.2)
Heroin	742 (75.9)	137 (58.8)	160 (74.4)	112 (80.0)	342 (85.7)
Street fentanyl	387 (39.6)	64 (27.5)	70 (32.6)	55 (39.3)	200 (50.0)
Opiate painkillers	509 (52.1)	96 (41.2)	90 (41.9)	79 (56.4)	249 (63.0)
*Proximity to SSP*					
Walking distance	470 (48.1)	84 (36.1)	100 (47.5)	73 (52.1)	213 (54.8)
< 30-min drive	412 (42.2)	113 (48.5)	95 (44.2)	58 (41.4)	146 (37.5)
> 30-min drive	78 (8.0)	30 (12.9)	17 (7.9)	8 (5.7)	23 (5.9)
No program reasonably close	12 (1.2)	6 (2.6)	1 (0.5)	1 (0.7)	4 (1.0)
Don’t know how close an SSP is	5 (0.5)	0 (0.0)	2 (0.9)	0 (0.0)	3 (0.8)

*SSP* Syringe service program, *SD* standard deviation, *SNAP* supplemental nutrition assistance program

^a^Reference period: prior 6 months

^b^Reference period: prior 30 days

**Table 4:** Unadjusted multinomial logistic regression associations between houselessness, covariates and the frequency of SSP use in the prior 30 days among Rural Opioid Initiative PWID *and* used an SSP at least once in the prior 30 days (*n* = 977)

Characteristic	Frequency of SSP use^b^					
	Twice versus once		Three times versus once		Four or more times versus once	
	OR	(95% CI)	OR	95% CI	OR	95% CI
Experienced houselessness^a^	0.96	(0.65, 1.43)	1.37	(0.87, 2.16)	**0.69**	**(0.49, 0.98)**
*Gender*^*c*^						
Man	Ref	–	Ref	–	Ref	–
Woman	1.01	(0.69, 1.50)	1.14	(0.75, 1.76)	0.95	(0.67, 1.33)
Age (years)	0.99	(0.97, 1.01)	0.96	(0.94, 0.98)	0.99	(0.97, 1.01)
*Race*^*d*^						
White	Ref	–	Ref	–	Ref	–
Native American	0.94	(0.45, 2.01)	1.43	(0.68, 3.01)	1.50	(0.80, 2.81)
Other	0.81	(0.37, 1.79)	0.87	(0.36, 2.13)	0.94	(0.47, 1.88)
*Ethnicity*						
Hispanic	0.55	(0.16, 1.88)	0.65	(0.17, 2.55)	1.20	(0.48, 3.00)
Graduated high school	1.35	(0.79, 2.32)	1.00	(0.57, 1.77)	0.68	(0.44, 1.05)
Received SNAP^a^	0.98	(0.66, 1.44)	0.88	(0.57, 1.36)	1.08	(0.76, 1.53)
Received food from food pantry^a^	1.12	(0.76, 1.66)	1.07	(0.70, 1.66)	0.91	(0.64, 1.29)
*Drug Use*^*b*^						
Methamphetamine/crystal	0.70	(0.38, 1.27)	0.92	(0.47, 1.80)	0.78	(0.45, 1.35)
Heroin	**2.11**	**(1.38, 3.22)**	**2.81**	**(1.69, 4.66)**	**3.77**	**(2.50, 5.68)**
Street fentanyl	1.28	(0.83, 1.99)	1.57	(0.98, 2.53)	**2.08**	**(1.41, 3.07)**
Opiate painkillers	1.04	(0.70, 1.52)	**1.90**	**(1.24, 2.93)**	**2.50**	**(1.76, 3.55)**
*Proximity to SSP*^*e*^						
Walking distance	Ref	–	Ref	–	Ref	–
< 30-min drive	0.74	(0.49, 1.11)	**0.59**	**(0.37, 0.94)**	**0.48**	**(0.33, 0.71)**
> 30-min drive	**0.48**	**(0.24, 0.94)**	**0.32**	**(0.13, 0.75)**	**0.33**	**(0.17 0.61)**
No program reasonably close	0.15	(0.02, 1.34)	0.22	(0.03, 1.94)	0.33	(0.08, 1.32)

Bold indicates statistically significant at *p *< 0.05

*SSP* syringe service program, *PWID* people who inject drugs, *OR* odds ratio, *CI* confidence intervals, *SNAP* supplemental nutrition assistance program

^a^Reference period: prior 6 months

^b^Reference period: prior 30 days

^c^Bivariate analysis for gender was performed excluding participants who reported an ‘other’ gender category (*n* = 5) because models would not converge due to small cell sizes

^d^The ‘Black’ racial category was combined with ‘other’ for the bivariate analysis of race because models would not converge due to small cell sizes

^e^Bivariate analysis for proximity to SSP was performed excluding participants who reported ‘don’t know how close an SSP is’ (*n* = 5) because models would not converge due to small cell sizes

**Table 5:** Adjusted multinomial logistic regression associations between houselessness and the frequency of SSP use in the prior 30 days among Rural Opioid Initiative PWID *and* used an SSP at least once in the prior 30 days (*n* = 972)

Characteristic	Frequency of SSP use^b^					
	Twice versus once		Three times versus once		Four or more times versus once	
	aOR	(95% CI)	aOR	95% CI	aOR	95% CI
Experienced houselessness^a^	0.90	(0.60, 1.36)	1.23	(0.77, 1.98)	**0.59**	**(0.40, 0.85)**
*Drug Use*^*b*^						
Heroin	**2.31**	**(1.43, 3.75)**	**2.47**	**(1.39, 4.40)**	**2.81**	**(1.75, 4.52)**
Street fentanyl	0.93	(0.56, 1.54)	0.94	(0.55, 1.60)	1.17	(0.74, 1.84)
Opiate painkillers	0.79	(0.51, 1.20)	1.43	(0.89, 2.29)	**1.77**	**(1.20, 2.61)**
*Proximity to SSP*						
Walking distance	Ref	–	Ref	–	Ref	–
< 30-min drive	0.70	(0.46, 1.07)	**0.60**	**(0.37, 0.96)**	**0.45**	**(0.31, 0.68)**
> 30-min drive	**0.49**	**(0.25, 0.99)**	**0.34**	**(0.14, 0.82)**	**0.33**	**(0.17, 0.65)**
No program reasonably close	0.16	(0.02, 1.44)	0.22	(0.02, 2.00)	0.39	(0.09, 1.65)

Bold indicates statistically significant at *p *< 0.05

*SSP* Syringe service program, *PWID* people who inject drugs, *aOR* adjusted odds ratios, CI confidence interval

Multivariable regression was performed excluding participants who reported ‘don’t know how close an SSP is’ (*n* = 5) because model would not converge due to small cell sizes

^a^Reference period: prior 6 months

^b^Reference period: prior 30 days

Compared to their housed counterparts, those who had experienced houselessness were just as likely to use an SSP two or three times compared to once in the prior 30 days (aOR = 0.90 [95% CI 0.60, 1.36] and aOR = 1.23 [95% CI 0.77, 1.98], respectively), as shown in Table 5. Participants who had experienced houselessness were less likely to use an SSP four or more times compared to once, relative to those who had not experienced houselessness in the prior 6 months (aOR = 0.59 [95% CI 0.40, 0.85]). Being further from an SSP was also associated with being less likely to use an SSP more frequently. Compared to those who lived within walking distance of an SSP, PWID who had to travel more than 30 min away were less likely to use it two (aOR = 0.49 [95% CI 0.25, 0.99]), three (aOR = 0.34 [95% CI 0.14, 0.82]), or four or more times (aOR = 0.33 [95% CI 0.17, 0.65]) compared to once. Similarly, those who lived less than 30 min from an SSP were less likely to use it three (aOR = 0.60 [95% CI 0.37, 0.96]) or four or more times (aOR = 0.45 [95% CI 0.31, 0.68]) compared to once, relative to those who could walk to an SSP.

## Discussion

This study extends research on the critical issue of houselessness and SSP use to rural areas, and provides evidence that rural US-based PWID who experience houselessness utilize SSPs at a similar or greater rate as their housed counterparts. PWID who experienced houselessness were 24% *more likely* to use an SSP at least once in the prior 30 days compared to housed PWID, and they were *just as likely* to use it two or three times compared to once. However, they were less likely to use an SSP four or more times. These findings are encouraging, since people who experience houselessness are at increased risk for multiple drug-related harms [2, 38, 39, 47, 57, 76–79]. The findings are also particularly striking in this sample of PWID who reside in rural environments that present unique challenges to accessing SSPs, especially among those experiencing houselessness (e.g., geographic dispersion of people and resources coupled with lack of public transportation).

The results of this study are congruent with the few studies that have examined the relationship between houselessness and SSP use elsewhere in the USA. A study in 23 US cities similarly found that PWID experiencing houselessness were 9% more likely to obtain syringes from an SSP in the past year compared to those who were not experiencing houselessness [35]. Another study in the state of Maine found that those experiencing houselessness were just as likely as their housed counterparts to use an SSP in the prior 3 months [14]. These results could be due to the implementation of more flexible harm reduction approaches in rural areas. For example, in many rural settings, SSPs provide mobile exchanges which may be particularly effective at reducing barriers (e.g., lack of transportation) that are especially prominent among those experiencing houselessness [80–82]. Rural people experiencing houselessness may also intentionally stay near areas where SSPs and other services are located for ease of access to resources [62, 83, 84]; conversely, SSPs may strategically open near places where people experiencing houselessness live or spend time. Our results could also indicate an increased need for various resources among unstably housed PWID. People experiencing houselessness may be more motivated to visit SSPs to not only obtain substance use-related resources, but also other resources that are vital to their well-being (e.g., food, clothing and referrals to other social services) [85, 86]. Research to identify how rural SSPs and PWID who experience houselessness are addressing and overcoming barriers to SSP use will be advantageous, providing SSPs an opportunity to implement targeted strategies to improve harm reduction service access among all rural PWID.

Despite these encouraging findings, our analyses also revealed that experiencing houselessness was associated with *reduced* likelihood of utilizing an SSP four or more times compared to once. This may indicate that visiting an SSP one to three times per month is sufficient for PWID who experience houselessness to meet their needs. However, an alternative explanation for this result—given that some studies have found that houselessness is associated with more frequent injection drug use [76, 87]—is that barriers related to housing instability impede consistent or more frequent SSP access. This is aligned with other studies that have found that PWID who experience houselessness *and* use SSPs are more likely to report inadequate syringe coverage (i.e., not having new syringes for each injection) [33, 36] and sharing injection equipment [35, 88] compared to their housed counterparts. The ability to frequently and consistently exchange a sufficient number of needles and syringes at SSPs is critical to harm reduction [89]. However, people experiencing houselessness may not use SSPs regularly or may exchange fewer syringes due to transportation challenges [90–92], inability to store syringes and/or inability to keep track of syringes to exchange, among others. For example, individuals who are more transient or are residing in public housing with drug-free policies may opt to visit SSPs less often because they lack a private, safe place to store new injection equipment [88]. Others may lose syringes or have them stolen, preventing them from acquiring new syringes at SSPs with strict one-for-one exchange policies. These findings highlight the need to understand and address the unique challenges that rural PWID and experience houselessness face to ensure that SSP exchange policies are equitable and that services are frequently utilized by those at high risk for drug-related harms. Future research should specifically explore SSP policies and practices to increase the implementation of need-based and secondary exchanges, which PWID have named as facilitators for safe injection practices [93].

A final important finding from this analysis is that SSP utilization was suboptimal among rural PWID, regardless of housing (in)stability. Less than half (44.0%) of ROI PWID reported using an SSP in the prior 30 days and in some areas as little as 10.6% used an SSP. Differences in utilization across ROI sites may partly be explained by the suspension and shut down of programs in some areas (e.g., West Virginia) [94] and the use of SSPs as primary sites of recruitment in others (e.g., Wisconsin). Two studies conducted in cities across the USA found that 53% and 65% of PWID reported SSP utilization in the prior 12 months [35, 95]. A study conducted in rural Maine found that 64% of PWID used SSPs in the prior 3 months [14], and another in rural West Virginia found that 68% of PWID used SSPs in the prior 6 months [33]. In our rural sample, PWID who lived within walking distance of an SSP were more likely to use its’ services, indicating that proximity to services will be critical to improve utilization. While SSPs have rapidly expanded to rural US areas in the last decade, our findings highlight the implications of unique challenges that the rural context presents to SSP utilization, including fewer SSPs [11, 15], limited SSP hours of operations and resources [11, 13, 96], geographic dispersion of resources and people [11, 91, 92, 95–97], and lack of public, affordable transportation [11, 13, 91, 92, 95]. Continued improvement of SSP access and utilization for rural PWID who are and are not unstably housed will be essential to mitigate drug-related epidemics.

### Strengths and limitations

The major strengths of this study come from the scope of the ROI study, which includes an unprecedented number of PWID from eight rural US areas in ten US states. The ROI study offers the most expansive, geographically diverse sample of rural PWID to date, to our knowledge [3]. However, this research is not without limitations. First, the ROI sample may not be representative of all rural PWID. For example, the ROI sample lacks racial diversity, which may or may not be representative of all US rural regions. Second, detailed information about houselessness was not captured, limiting our ability to know when houselessness occurred in the prior 6 months (i.e., participants may not have experienced houselessness in the prior 30 days, which is the recall period for SSP use) and whether it was persistent. Regardless, experiencing houselessness to any degree within a 6-month period is likely indicative of broader experiences of housing instability, which has been shown to be associated with drug-related harms [34, 67–69] and access to health services [70, 71]. Lastly, the survey did not ask participants why they did or did not use an SSP in the prior 30 days, whether there were barriers to use or what services they received. Research is needed to understand these nuances to develop approaches to best serve PWID.

## Conclusions

This study expands upon the limited research on rural SSP use, providing insights into utilization among PWID who are and are not unstably housed. Findings revealed that SSP use was generally low among ROI PWID, but those who had experienced houselessness were more likely to report using an SSP at least once in the prior 30 days. These results are encouraging as people who experience houselessness are at increased risk for multiple drug-related harms and may encounter additional challenges when attempting to access SSPs. Future research to identify how PWID who experience houselessness overcome barriers and utilize SSPs in rural contexts could offer insights to expand harm reduction service access among all rural PWID.

### Supplementary Information

**Additional file 1: Table S1.** Overall and site-specific SSP use and houselessness among people who injected drugs enrolled in the Rural Opioid Initiative (*n* = 2394). **Table S2. **Overall and site-specific frequency of SSP use by people who injected drugs who used an SSP at least once in the prior 30 days enrolled in the Rural Opioid Initiative (*n *= 977).

## Data Availability

We welcome collaboration and encourage mentorship and the use of ROI data stripped of all protected health information (PHI) to enable early investigators to address meaningful questions with support to help ensure their success. Additional information can be obtained at the ROI website: ruralopioidinitiative.org or by contacting the ROI DCC at ruralopioidinitiative@uw.edu. Follow the Rural Opioid Initiative on Twitter @ruralopioids.

## Abbreviations

AOR: Adjusted odds ratio
CI: Confidence interval
HCV: Hepatitis C virus
HIV: Human immunodeficiency virus
OR: Odds ratio
PWID: People who inject drugs
RDS: Respondent-driven sampling
ROI: Rural Opioid Initiative
SD: Standard deviation
SNAP: Supplemental nutrition assistance program
SSP: Syringe service program
USA: United States of America
