# Supplementary material for: Houselessness and syringe service program utilization among people who inject drugs in eight rural areas across the USA: a cross-sectional analysis
Source: Harm Reduct J. 2023 Oct 26;20:157. doi: 10.1186/s12954-023-00892-w (PMC10601138; doi:10.1186/s12954-023-00892-w)
Supplement: Supplementary file 1 — Additional file 1: Table S1. Overall and site-specific SSP use and houselessness among people who injected drugs enrolled in the Rural Opioid Initiative (n = 2394). Table S2. Overall and site-specific frequency of SSP use by people who injected drugs who used an SSP at least once in the prior 30 days enrolled in the Rural Opioid Initiative (n = 977). [file 12954_2023_892_MOESM1_ESM.docx]

**Houselessness and syringe service program utilization among people who inject drugs in eight rural areas across the United States: A cross-sectional analysis**

**Additional File 1**

April M Ballard, PhD^1,2*^; Dylan Falk, MPH^1^; Harris Greenwood, MS, MPH^1^; Paige Gugerty, MPH^1^; Judith Feinberg^3^; Peter D Friedmann^4^; Vivian F Go^5^; Wiley D Jenkins^6^; P Todd Korthuis^7^; William C Miller^8^; Mai T Pho^9^; David W Seal^10^; Gordon S Smith^3^; Thomas J Stopka^11^; Ryan P Westergaard^12^; William A Zule^13^; April M Young, PhD^14^; Hannah LF Cooper, ScD, SM^1^

1. Rollins School of Public Health, Emory University, 1518 Clifton Road, Atlanta, Georgia 30322
2. School of Public Health, Georgia State University, 140 Decatur Street SE, Atlanta, Georgia 30303
3. School of Medicine, West Virginia University, Morgantown, WV, USA
4. Office of Research, University of Massachusetts Chan Medical School-Baystate, Springfield, Massachusetts, USA
5. Gillings School of Global Public Health, University of North Carolina at Chapel Hill, Chapel Hill, NC, USA
6. School of Medicine, Southern Illinois University, Carbondale, Illinois, USA
7. Department of Medicine, Oregon Health & Science University, Portland, Oregon, USA
8. College of Public Health, The Ohio State University, Columbus, Ohio, USA
9. Department of Medicine, University of Chicago, Chicago, Illinois, USA
10. Tulane University School of Public Health and Tropical Medicine, New Orleans, Louisiana, USA
11. School of Medicine, Tufts University, Boston, Massachusetts, USA
12. School of Medicine and Public Health, University of Wisconsin-Madison, Madison, Wisconsin, USA
13. RTI International, Research Triangle Park, North Carolina, USA
14. College of Public Health, University of Kentucky, 111 Washington Avenue, Lexington, Kentucky 40536

***Corresponding Author:** Email: [aballard11@gsu.edu](mailto:aballard11@gsu.edu)

**Table S.1.** Overall and site-specific SSP use and houselessness among people who injected drugs enrolled in the Rural Opioid Initiative (*n*=2,394)

|  | n | Percent who used an SSP^a^ | Percent who experienced houselessness |
| --- | --- | --- | --- |
| Overall | 2394 | 43.5 | 56.1 |
| Illinois | 122 | 12.3 | 55.7 |
| Kentucky | 244 | 43.4 | 38.1 |
| North Carolina | 280 | 36.1 | 45.0 |
| New England | 409 | 26.4 | 57.9 |
| Ohio | 197 | 49.7 | 57.4 |
| Oregon | 148 | 37.2 | 68.2 |
| Wisconsin | 863 | 63.2 | 62.7 |
| West Virginia | 131 | 10.7 | 48.9 |

1. Reference period: prior 30 days

**Table S.2.** Overall and site-specific frequency of SSP use by people who injected drugs who used an SSP at least once in the prior 30 days enrolled in the Rural Opioid Initiative (*n*=977)

| Characteristics |  | Frequency of SSP use in prior 30 days | | | |
| --- | --- | --- | --- | --- | --- |
|  | **Total** | **Once** | **Twice** | **Three times** | **Four or more times** |
|  | **n (%)** | **n (%)** | **n (%)** | **n (%)** | **n (%)** |
|  | 977 (100.0) | 233 (23.8) | 215 (22.0) | 140 (14.3) | 389 (39.8) |
| ROI study site |  |  |  |  |  |
| Illinois | 15 (1.5) | 4 (1.7) | 5 (2.3) | 1 (0.7) | 5 (1.3) |
| Kentucky | 106 (10.8) | 30 (12.9) | 21 (9.8) | 15 (10.7) | 40 (10.3) |
| North Carolina | 101 (10.3) | 17 (7.3) | 7 (3.3) | 15 (10.7) | 62 (15.9) |
| New England | 108 (11.1) | 19 (8.2) | 24 (11.2) | 12 (8.6) | 53 (13.6) |
| Ohio | 98 (10.0) | 9 (3.9) | 17 (7.9) | 16 (11.4) | 56 (14.4) |
| Oregon | 54 (5.5) | 22 (9.4) | 14 (6.5) | 5 (3.6) | 13 (3.3) |
| Wisconsin | 481 (49.2) | 126 (54.1) | 126 (58.6) | 75 (53.6) | 154 (39.6) |
| West Virginia | 14 (1.4) | 6 (2.6) | 1 (0.5) | 1 (0.7) | 6 (1.5) |
